# Future anxiety, depression and stress among undergraduate students: psychological flexibility and emotion regulation as mediators

**DOI:** 10.3389/fpsyg.2025.1517441

**Published:** 2025-01-31

**Authors:** Gülçin Güler Öztekin, Juan Gómez-Salgado, Murat Yıldırım

**Affiliations:** ^1^Department of Psychology, Faculty of Science and Letters, Agri Ibrahim Cecen University, Ağrı, Türkiye; ^2^Department of Sociology, Social Work and Public Health, Faculty of Labour Sciences, University of Huelva, Huelva, Spain; ^3^Safety and Health Postgraduate Program, Universidad Espíritu Santo, Guayaquil, Ecuador; ^4^Psychology Research Centre, Khazar University, Baku, Azerbaijan

**Keywords:** future anxiety, psychological flexibility, cognitive reappraisal, expressive suppression, depression, stress

## Abstract

**Introduction:**

Mental health and wellbeing are fundamental and integral components of healthy functioning, and psychological resources significantly contribute to its maintenance and enhancement. This study aimed to investigate the mediating effects of psychological flexibility and emotion regulation in the association between future anxiety, depression, and stress.

**Methods:**

A total of 528 undergraduate students participated in this study (*M* = 20.14, *SD* = 1.76).

**Results:**

The findings of the study showed that future anxiety was negatively associated with psychological flexibility and cognitive reappraisal, and positively associated with expressive suppression, depression and stress. Psychological flexibility and cognitive reappraisal had negative relationships with depression and stress, and expressive suppression had a positive relationship with depression and stress. The associations between future anxiety, depression and stress were mediated by psychological flexibility, cognitive reappraisal and expressive suppression.

**Discussion:**

This study deepened our understanding of the possible mechanisms of depression and stress. For individuals suffering from future anxiety, psychological flexibility and emotion regulation may be a coping strategy that leads individuals to less depression and stress. This study suggests that psychological flexibility and emotion regulation skills are fundamental aspects of psychological health.

## Introduction

Mental health and wellbeing are fundamental and integral components of healthy functioning, and psychological resources significantly contribute to its maintenance and enhancement (Masoom Ali et al., 2020; Yıldırım, 2020; Yıldırım et al., 2023a). A portion of the global burden of disease is due to neuropsychiatric disorders because of the chronically damaging nature of depression and other common mental disorders, alcohol and substance use disorders, and psychoses (Prince et al., 2007). Depression, anxiety and stress are mental health problems that are frequently experienced by individuals and significantly affect their wellbeing and health (Karyotaki et al., 2020; Rashid et al., 2023; Yıldırım and Özaslan, 2022). Depression refers to a mental health disorder that occurs in a person’s life, with symptoms including sad mood, somatic complaints, loss of interest, regression, pessimism, worthlessness, and guilt (Fried, 2017). Depression negatively affected the indicators of psychological wellbeing: autonomy, environmental mastery, personal growth, positive relations with others, purpose in life, and self-acceptance (Strauss et al., 2012). Depressed individuals had a low quality of life (Mei et al., 2021) and poor life satisfaction (Li et al., 2021). These individuals adopted maladaptive coping strategies such as behavioral disengagement, denial, self-blame, self-distraction, and substance use rather than adaptive coping strategies such as active coping, planning, and positive reframing (Lopes and Nihei, 2021). Depression also increased the risk of many problems such as sleep problems (Morssinkhof et al., 2020), eating disorder (Sander et al., 2021), cardiovascular disease (Kwapong et al., 2023), and even suicidal ideation, suicide attempts and suicide death (Li et al., 2022). Therefore, depression is not a temporary state of sadness but is considered a serious mental health problem that may require professional intervention.

Stress is a complex phenomenon that leads to mental health disorders and chronic health conditions, and reduces productivity and quality of life. “Eustress,” a positive psychological response to a stressor, is beneficial because it motivates the individual to cope with the demands, but chronic and prolonged stress overwhelms the body’s coping mechanisms (Manosso et al., 2022). Stress had deleterious effects on mental wellbeing, subjective wellbeing and psychological wellbeing (Lee, 2017). Stress caused a weakened immune system (Lueke and Assar, 2024), sleep problems, mental disorders (Kaur et al., 2021), and heart diseases (Yin et al., 2021). The ability to cope with depression and stress is critical for individuals to maintain their health and live a healthy life. Therefore, factors that lead to depression and stress should be identified for the mental health of both individuals and societies.

Future anxiety refers to a state of worry, uncertainty, fear and concern about negative changes in the future. In an extreme case, this could be a threat in which something catastrophic may happen to a person. More importantly, future anxiety involves subjective states where the personal future is at stake. Although the fear experienced is clear and conscious, it is due to cognitive representations of the future rather than actual events. In other words, the fear is experienced here and now but refers to future events (Zaleski, 1996). Future anxiety and pessimism, while related, are distinct concepts. Future anxiety refers to a sense of uncertainty about what lies ahead, which can restrict individuals in their planning and decision-making processes (Zaleski, 1996). In contrast, pessimism is more self-oriented and involves a perception that negative outcomes are inevitable. It represents a deeply ingrained mindset, where individuals consistently believe that unfavorable events will persist in the future (Scheier et al., 2001). Future anxiety undermined self-efficacy (Rabei et al., 2020), quality of life (Kästner et al., 2022), and decision-making skills (Al Hwayan, 2020). Future anxiety was identified as a factor of sleep disturbances that led to risky behaviors such as suicide and psychological problems (Ahmad et al., 2024; Baldini et al., 2024). This anxiety contributed to loneliness (Jannini et al., 2024), behavioral addictions (Pan et al., 2024), and psychological distress (Dey et al., 2022). In particular, anxiety about the future was related to general malaise and considered a risk factor for depression and stress (Regnoli et al., 2024a). As can be understood from these studies, anxiety about the future can negatively affect psychological health and lead to depression and stress. Determining protective factors to reduce the negative effects of this relationship is necessary for the mental health of individuals.

### The mediating roles of psychological flexibility and emotion regulation

Psychological flexibility refers to the ability to connect with the present moment by completely distancing oneself from past and future concerns; this occurs through the ability to actively, openly, and nonjudgmentally embrace inner experiences and the reduced tendency to control these inner experiences. This also refers to the ability of a person to persist in or modify behavior toward the attainment of chosen goals or values (Bond et al., 2008; Hayes et al., 2006). A growing body of research has highlighted the numerous benefits of psychological flexibility for individuals (Tanhan et al., 2024; Yıldırım et al., 2024a, 2024b). For example, increasing the psychological flexibility ability of individuals decreases emotional exhaustion (Lloyd et al., 2013) and future anxiety (Hasan Alam et al., 2023), and increased life satisfaction (Lucas and Moore, 2020) and wellbeing (Wersebe et al., 2018). Psychological flexibility contributed significantly to enhancing psychological wellbeing (Guerrini Usubini et al., 2021). However, low psychological flexibility led to poor psychological health and emotional distress (Masuda et al., 2010). In particular, this poor flexibility ability contributed to somatization, depression, anxiety, and general distress (Masuda and Tully, 2012).

Previous studies have also provided evidence of the mediating role of psychological flexibility. Psychological flexibility acted as a mediator in the association between health anxiety and psychological distress (Landi et al., 2020). Psychological flexibility buffered the adverse relationship between symptoms and functioning (Gentili et al., 2019). Psychological flexibility mediated the relationship between self-concealment and emotional distress and general psychological ill-health in stressful interpersonal environments (Masuda and Tully, 2012). Psychological inflexibility mediated the relationship between anxiety, depression, and emotional eating (Guerrini Usubini et al., 2022b). These studies highlight the protective role of psychological flexibility.

Emotion regulation refers to a process by which individuals modify their emotions, their reactions to emotions, or the situations that elicit emotions to respond appropriately to environmental demands (Gross, 1998). Two specific strategies for emotion regulation are cognitive reappraisal and expressive suppression. Cognitive reappraisal can be defined as the attempt to reinterpret an emotionally arousing situation in a way that changes its emotional impact (Gross and John, 2003; Lazarus and Alfert, 1964). Expressive suppression can be defined as an attempt to inhibit ongoing emotion-expressive behavior (Gross, 1998). Many studies have shown that greater use of cognitive reappraisal strategies contributed to resilience and better mental health (Cardi et al., 2021) and frequent use of expressive suppression strategies led to increased psychological symptoms, poor mental health and low wellbeing (Chervonsky and Hunt, 2019). Social anxiety and anxiety sensitivity had a positive relationship with emotion regulation difficulties (Paulus et al., 2016; Sertbaş et al., 2020). On the other hand, low emotion regulation levels led to psychological distress such as depression and stress (Berking et al., 2014; Seligowski et al., 2015) and impulsive behaviors (Alfonsi et al., 2023; Guerrini Usubini et al., 2022a). Previous studies have also indicated the mediator role of emotion regulation. For example, the link between attachment anxiety and depression was mediated by emotion regulation (Colonnello et al., 2022). Heightened use of cognitive reappraisal and dampened use of expressive suppression served as mediators in the relationship between beliefs about emotion controllability and psychological health (Deplancke et al., 2023). These studies indicate the positive effects of cognitive reappraisal and the negative effects of expressive suppression on mental health.

University students are plagued by higher uncertainty and less permanence in their current lives and also in the anticipated future. Rabei et al. (2020) reported that students’ future anxiety increased throughout their school life, especially in the last years of their education. Future anxiety is increasingly emerging among university students not only from the fear of failing their studies but also from the fear of lack of job opportunities. Hammad (2016) declared that this affects these students’ participation in their specialization. In this regard, probable future events increase the possibility of experiencing anxiety and depression (Eysenck et al., 2006). Given that students cope with this anxiety during their university years, which significantly impacts their career plans and psychological wellbeing, identifying factors that can mitigate these negative effects is important for safeguarding both their mental health and future prospects. Based on the theoretical framework and empirical evidence documented above, we propose that psychological flexibility and cognitive reappraisal may reduce the relationship between future anxiety, depression and stress, while expressive suppression may foster this relationship.

### Present study

The above-mentioned previous literature has identified a positive relationship between future anxiety, depression and stress. However, to our knowledge, there is no study on the mediation of psychological flexibility, cognitive reappraisal, and expressive suppression in this relationship. Therefore, this study aimed to examine the mediation roles of psychological flexibility, cognitive reappraisal, and expressive suppression in the relationship between future anxiety, depression and stress. To this end, we proposed the following hypotheses:

H1: Future anxiety will have a negative association with psychological flexibility and cognitive reappraisal, and a positive association with expressive suppression, depression and stress.

H2: Psychological flexibility and cognitive reappraisal will be negatively related to depression and stress, and expressive suppression will be positively related to depression and stress.

H3: Psychological flexibility, cognitive reappraisal, and expressive suppression will mediate the association between future anxiety depression and stress.

**Figure 1:**
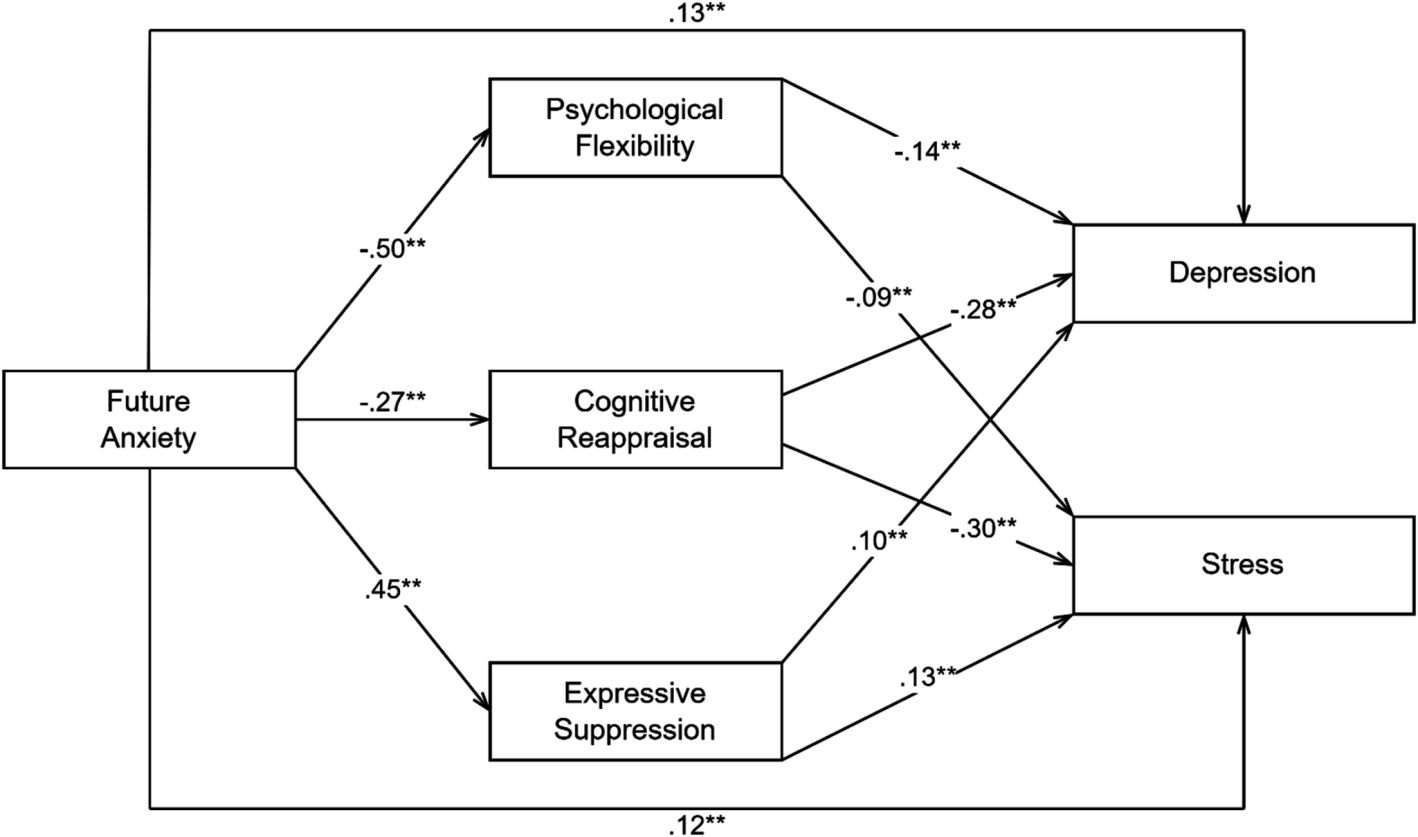
The conceptual model.

The conceptual model is illustrated in Figure 1.

## Method

### Participants

Participants included 528 undergraduate students. 59.7% of the participants (*n* = 315) were women and 40.3% were men. The students’ ages ranged from 18 to 28, with a mean age of 20.14 years (*SD* = 1.76). 181 (34.3%) of the students were freshmen, 158 (29.9%) were sophomores, 108 (20.5%) were juniors, and 81 (15.3%) were senior students. Among the participants, 20.5% perceived their household income as low, 68% as middle, and 11.5% as high.

### Measures

#### Dark future scale

This scale was developed by Zaleski et al. (2019) and adopted to Turkish culture by Yıldırım et al. (2023b) to measure concern and anxiety toward the future. The scale is unidimensional and consists of five items. Participants respond on a 6-point Likert scale ranging from 1 (decidedly false) to 6 (decidedly true). The sample item of the scale is “I am afraid that the problems which trouble me now will continue for a long time.” High scores indicate greater levels of future anxiety. The Cronbach’s alpha coefficient was 0.79. In the current study, the Cronbach’s alpha coefficient was calculated as 0.89.

#### Psy-flex scale

This scale was developed by Gloster et al. (2021) and adopted to Turkish culture by Yıldırım and Aziz (2023) to assess the psychological flexibility levels of individuals. The scale is unidimensional and consists of six items. Participants respond on a 5-point Likert scale ranging from 1 (very seldom) to 5 (very often). The sample item of the scale is “I am completely interested in things that are important, useful or meaningful to me.” High scores reflect higher levels of psychological flexibility. The Cronbach’s alpha coefficient was calculated as 0.80. In the current study, the Cronbach’s alpha coefficient was 0.85.

#### Emotion regulation questionnaire-short form (ERQ-S)

This scale was developed by Preece et al. (2023) and adopted to Turkish culture by Dilekçi and Yıldırım (submitted for publication) to measure the emotion regulation levels of individuals. The scale consists of two subscales with six items: cognitive reappraisal and expressive suppression. Participants score on a 7-point Likert scale ranging from 1 (strongly disagree) to 7 (strongly agree). The sample item of the scale is “I control my emotions by not expressing them.” High scores show higher cognitive reappraisal and expressive suppression levels. Adequate evidence of Cronbach’s alpha has been reported in previous research (Dilekçi and Yıldırım, submitted for publication; Preece et al., 2023). In the current study, the Cronbach’s alpha coefficients were 0.86 and 0.81, respectively.

#### Depression, stress and anxiety scale (DASS21)-short form

This scale was developed by Lovibond and Lovibond (1995) and adopted to Turkish culture by Yılmaz et al. (2017) to assess depression, anxiety and stress levels of individuals. The scale consists of three subscales with 21 items: depression, anxiety and stress. Participants score on a 4-point Likert scale ranging from 0 (not suitable for me) to 3 (completely suitable for me). The sample item of the scale is “I could not stand things that distracted me from what I was doing.” High scores indicate higher levels of depression, anxiety and stress. In this study, the depression and stress sub-dimensions of the scale were applied. The Cronbach’s alpha coefficient was calculated as 0.81 for depression and 0.75 for stress. In the current study, Cronbach’s alpha coefficients were 0.80 and 0.79 for depression and stress, respectively.

### Procedure

Necessary ethical permission was obtained from the Institutional Review Board and the Declaration of Helsinki guidelines were followed (Date: 26 September 2024, Ethic Code: E-95531838-050.99-112421). Data was collected via a web-based survey. The online survey form was sent to university students using the snowball sampling method. Participants were informed about their rights before, during and after participation. Participation was completely voluntary. Informed consent was obtained from all participants, and they were subsequently invited to participate in the survey. Anonymity and confidentiality were ensured, and no compensation was provided.

### Statistical analyses

First, descriptive statistics were conducted. Normality was evaluated using kurtosis and skewness values, with acceptable ranges falling between +1.5 and −1.5 (Tabachnick et al., 2013). Then, correlation analysis was performed to examine the relationship between the study variables. Finally, mediation analyses were performed using Hayes’ PROCESS-Macro v4.2 (Model 4). To investigate indirect mediation effects, bootstrapping was applied with 5,000 resampling and 95% confidence intervals with bias correction (Hayes, 2009). All data analyses were performed with SPSS version 27 for Windows.

## Results

The preliminary analysis results are presented in Table 1. Skewness values were between 0.41 and −0.18, kurtosis values were between 0.01 and −1.21. These values indicated that the normality assumption was met. Correlation analysis results showed that future anxiety had significant positive correlations with expressive suppression, depression and stress, and significant negative correlations with psychological flexibility and cognitive reappraisal. Psychological flexibility and cognitive reappraisal had significant negative correlations with depression and stress. Expressive suppression had significant positive correlations with depression and stress.

**Table 1:** Descriptive statistics, skewness, kurtosis, and correlations.

Variables	*M*	*SD*	Skewness	Kurtosis	Correlation					
					1	2	3	4	5	6
1. Future anxiety	18.37	6.01	−0.18	−0.92	–					
2. Psychological flexibility	17.81	5.13	−0.07	−0.31	−0.58**	–				
3. Cognitive reappraisal	12.85	4.58	−0.09	−1.21	−0.35**	0.59**	–			
4. Expressive suppression	11.66	4.53	0.09	−1.05	0.59**	−0.40**	−0.54**	–		
5. Depression	8.73	3.85	0.14	−0.31	0.51**	−0.56**	−0.59**	0.50**	–	
6. Stress	8.94	3.68	0.41	0.01	0.50**	−0.53**	−0.61**	0.53**	0.64**	–

M, mean; SD, standard deviations; **p < 0.001.

Table 2 presents the results of the mediation analyses. Future anxiety significantly predicted psychological flexibility (*β* = −0.50, *p* < 0.001), cognitive reappraisal (*β* = −0.27, *p* < 0.001), expressive suppression (*β* = 0.45, *p* < 0.001), depression (*β* = 0.13, *p* < 0.001), and stress (*β* = 0.12, *p* < 0.001). Future anxiety explained 34% of the variance in psychological flexibility, 13% of the variance in cognitive reappraisal and 35% of the variance in expressive suppression. Psychological flexibility (*β* = −0.14, *p* < 0.001), cognitive reappraisal (*β* = −0.28, *p* < 0.001) and expressive suppression (*β* = 0.10, *p* < 0.001) significantly predicted depression, explaining 48% of the variance. Psychological flexibility (*β* = −0.09, *p* < 0.001), cognitive reappraisal (*β* = −0.30, *p* < 0.001) and expressive suppression (*β* = 0.13, *p* < 0.001) significantly predicted stress, explaining 49% of the variance.

**Table 2:** Unstandardized coefficients for the mediation model.

Consequent																								
Antecedent		M_1_ (Psychological flexibility)					M_2_ (Cognitive reappraisal)					M_3_ (Expressive suppression)					Y_1_ (Depression)				Y_2_ (Stress)			
		Coeff.	SE	*t*	*p*		Coeff.	SE	*t*	*p*		Coeff.	SE	*t*	*p*		Coeff.	SE	*t*	*p*	Coeff.	SE	*t*	*p*
X (Future anxiety)	a_1_	−0.50	0.03	−16.65	0.00	a_2_	−0.27	0.03	−8.82	0.00	a_3_	0.45	0.02	17.04	0.00	c’	0.13	0.02	4.54	0.00	0.12	0.02	4.35	0.00
M_1_ (Psychological flexibility)		–	–	–	–		–	–	–	–		–	–	–	–	b_1_	−0.14	0.03	−4.09	0.00	−0.09	0.03	−2.96	0.00
M_2_ (Cognitive reappraisal)		–	–	–	–		–	–	–	–		–	–	–	–	b_2_	−0.28	0.03	−7.79	0.00	−0.30	0.03	−8.51	0.00
M_3_ (Expressive suppression)		–	–	–	–		–	–	–	–		–	–	–	–	b_3_	0.10	0.03	2.73	0.00	0.13	0.03	3.65	0.00
Constant	i_M1_	27.04	0.58	46.40	0.00	i_M2_	17.88	0.59	29.83	0.00	i_M3_	3.38	0.51	6.63	0.00	i_y_	11.29	0.92	12.15	0.00	10.76	0.88	12.21	0.00
		*R*^2^ = 0.34					*R*^2^ = 0.13					*R*^2^ = 0.35					*R*^2^ = 0.48				*R*^2^ = 0.49			
		*F* = 277.50; *p* < 0.001					*F* = 77.95; *p* < 0.001					*F* = 290.64; *p* < 0.001					*F* = 120.71; *p* < 0.001				*F* = 124.65; *p* < 0.001			

X = independent variable, M = mediator variable, Y = dependent variable, Coeff. = unstandardized coefficient, SE = standard error.

Table 3 showed that future anxiety had a direct effect on depression (effect = 0.13, [0.07, 0.19]), and future anxiety had an indirect effect on depression through psychological flexibility (effect = 0.07, [0.02, 0.12]), cognitive reappraisal (effect = 0.08, [0.04, 0.11]) and expressive suppression (effect = 0.04, [0.01, 0.08]). Future anxiety had a direct effect on stress (effect = 0.12, [0.06, 0.17]), and future anxiety had an indirect effect on stress through psychological flexibility (effect = 0.04, [0.01, 0.09]), cognitive reappraisal (effect = 0.08, [0.05, 0.11]) and expressive suppression (effect = 0.05, [0.02, 0.10]).

**Table 3:** Total, direct, and indirect effects.

	Effect	*SE*	BootLLCI	BootULCI
Path 1				
Future anxiety → Psychological flexibility → Depression	0.07	0.02	0.02	0.12
Future anxiety → Cognitive reappraisal → Depression	0.08	0.01	0.04	0.11
Future anxiety → Expressive suppression → Depression	0.04	0.02	0.01	0.08
Total indirect effect	0.19	0.02	0.14	0.25
Direct effect	0.13	0.02	0.07	0.19
Total effect	0.33	0.02	0.28	0.37
Path 2				
Future anxiety → Psychological flexibility → Stress	0.04	0.02	0.01	0.09
Future anxiety → Cognitive reappraisal → Stress	0.08	0.01	0.05	0.11
Future anxiety → Expressive suppression → Stress	0.05	0.01	0.02	0.10
Total indirect effect	0.18	0.02	0.14	0.23
Direct effect	0.12	0.02	0.06	0.17
Total effect	0.31	0.02	0.26	0.35

Number of bootstrap samples for percentile bootstrap confidence intervals: 5.000.

## Discussion

University students need to have good mental health for their academic life, their general life as well as their future. The findings of the present study showed that future anxiety had an inverse relationship with psychological flexibility and cognitive reappraisal, and a positive relationship with expressive suppression, depression and stress. University students experiencing future anxiety reported low psychological flexibility and cognitive reappraisal levels, whereas high expressive suppression, depression and stress levels. Consistent with our results, Hasan Alam et al. (2023) found a relationship between decreasing future anxiety and increasing psychological flexibility. Szota et al. (2024) revealed that future anxiety exacerbated depression. A negative attitude toward the future or a negative representation of the future leads to depression and stress and is a risk factor for mental suffering (Regnoli et al., 2024b). Anxiety appeared to be associated with less use of cognitive reappraisal and greater use of expressive suppression (Dryman and Heimberg, 2018). These findings indicate the adverse effects of future anxiety on individuals in many areas such as psychological, cognitive and emotional functions.

The current study showed that psychological flexibility and cognitive reappraisal were negatively related to depression and stress, and expressive suppression was positively related to depression and stress. Participants who were psychologically flexible and regulated their emotions using cognitive reappraisal reported lower levels of depression and stress, whereas participants who used expressive suppression reported higher levels of depression and stress. There are studies in existing literature that support these results. For example, low psychological flexibility was associated with higher general distress (Kroska et al., 2020). Psychological flexibility was also identified as a resilience factor in individuals experiencing chronic pain and psychological distress (Gentili et al., 2019). On the other hand, emotion regulation difficulties increase depression and stress (Honan et al., 2023). In particular, cognitive reappraisal was negatively associated with health anxiety, but expressive suppression was positively associated with this anxiety (Akbari et al., 2021). A systematic review study found that cognitive reappraisal had an inverse relationship with depression, and expressive suppression had a positive relationship with depression (Dryman and Heimberg, 2018). These studies show that psychological flexibility and cognitive reappraisal contribute positively to the reduction of depression and stress, while expressive suppression contributes to the increase of these psychological distress.

The results of the present study also determined the mediating roles of psychological flexibility, cognitive reappraisal, and expressive suppression in the link between future anxiety, depression and stress. University students with high future anxiety reported low psychological flexibility and cognitive reappraisal levels and high expressive suppression levels, which in turn led to high depression and stress. Similarly, in individuals with pandemic-related concerns, psychological flexibility reduced psychological distress by reducing avoidant coping and increasing approach coping strategies (Tindle et al., 2022). Psychological flexibility mediated the relationship between negative early trauma effects and symptoms (Richardson and Jost, 2019) and between temperament and symptoms of stress and depression (Puolakanaho et al., 2023). The association between health anxiety and COVID-19 distress, anxiety, and depression was also mediated by psychological flexibility (Landi et al., 2020). In addition, cognitive reappraisal mediated the relationship between trait anxiety and expressive flexibility, but expressive suppression failed to mediate these associations (Shangguan et al., 2024). Emotion regulation buffered the deleterious effect of anxiety sensitivity, which plays an important role in the development and maintenance of anxiety symptoms, on depression (Ouimet et al., 2016). These results suggest that psychological flexibility and cognitive reappraisal are powerful assets that reduce the risk of mental health problems, while expressive suppression is a factor that exaggerates these problems.

The findings of this study contribute to the body of knowledge in literature on how to support the mental health of university students. The current results also offer several practical implications. A study conducted in Türkiye determined that factors such as employment anxiety, anxiety about not being appointed, stress about the exam required for appointment, difficulty of courses, inability to adapt, economic problems, hopelessness, difficulty of life, wrong department choice and high number of graduates were related to future anxiety (Esmer and Arıbaş, 2023). In addition, the current study determined a positive relationship between future anxiety, depression and stress. These results emphasize the necessity of dealing with these sources of anxiety to protect and improve the psychological health of university students. In this regard, creating awareness programs, organizing events for students to recognize their strengths and weaknesses, guiding them to acquire professional skills specific to their fields, and organizing development-oriented events that can help them create future plans in areas such as time management, problem-solving, goal setting, and creativity can be helpful for these students at universities. Furthermore, this study revealed that psychological flexibility and emotion regulation mediated the relationship between future anxiety, depression and stress. People have the potential to tolerate and effectively use emotions, thoughts, and behaviors to achieve the best possible outcomes in changing situations. These dynamic capabilities form the building blocks of health. In addition, a healthy person is aware of the possibility of novelty and change and who can manage themselves in the uncertain, unpredictable world around them (Kashdan and Rottenberg, 2010). In many forms of psychopathology, such as depression, anxiety, and stress, these flexibility processes and emotion regulation skills are deficient (Fischer et al., 2016; Lincoln et al., 2022). Therefore, to support mental health, mental health professionals should integrate practices aimed at increasing psychological flexibility and emotion regulation into their interventions. Specifically, these interventions should include practices such as helping individuals recognize, understand, control, and effectively express their emotions, reframing thoughts, recognizing automatic thoughts, coping with stress and challenges, identifying alternative routes, self-acceptance, and future-oriented thinking.

Although this study provides valuable contributions to existing literature, there are important limitations that should be considered when interpreting the study findings. The cross-sectional, correlational nature of the data was a limitation that prevented robust conclusions about directionality. Despite the anonymous nature of data collection, reliability was likely subject to social desirability and demand artifacts because the data were collected using self-report measures. Data were collected from undergraduate students at a public university, which limits the generalizability of the results to a larger population.

In conclusion, to benefit from the many advantages offered by university years, psychological support for students at this educational level should be a priority. The present study found that future anxiety was negatively related to psychological flexibility and cognitive reappraisal, and positively related to expressive suppression, depression and stress. Psychological flexibility and cognitive reappraisal were negatively associated with depression and stress, and expressive suppression was positively associated with depression and stress. Effective psychological flexibility and emotion regulation through increased use of cognitive reappraisal and reduced use of expressive suppression served as a mechanism to protect against the negative impact of future anxiety, depression, and stress. In this regard, the need to improve the psychological flexibility and emotion regulation skills of university students has emerged to achieve better mental health outcomes.

## Data Availability

The raw data supporting the conclusions of this article will be made available by the authors without undue reservation.
